# A Strange Case of Fever: Hematopyometra As the Initial Presentation of Endometrial Adenocarcinoma

**DOI:** 10.7759/cureus.84278

**Published:** 2025-05-17

**Authors:** Andreia Mandim, Ana Sofia Silva, Rita Moça, Luciana Faria, Fani Ribeiro, Rubina Silva

**Affiliations:** 1 Internal Medicine, Centro Hospitalar Póvoa de Varzim/Vila do Conde, Póvoa de Varzim, PRT

**Keywords:** endometrial adenocarcinoma, hematometra, postmenopausal bleeding, pyometra, uterine neoplasms

## Abstract

Pyometra is a rare condition involving the accumulation of purulent material within the uterine cavity, typically affecting postmenopausal women. Clinical presentation varies and may be silent, but can include fever, abdominal pain, and vaginal discharge. In this case, we report a 62-year-old woman presenting with prolonged constitutional symptoms and fever, ultimately diagnosed with a large hematopyometra secondary to endometrial adenocarcinoma. This case emphasizes the need to consider gynecological causes in elderly female patients with persistent systemic symptoms.

## Introduction

Pyometra is a rare gynecological condition with an estimated incidence below 1% in the general gynecologic population [1]. It predominantly affects elderly, postmenopausal women and may present with non-specific symptoms such as lower abdominal pain, vaginal discharge, and postmenopausal bleeding [2]. However, many cases remain asymptomatic until complications develop. Obstruction of cervical drainage, often due to malignancy, is the most common etiology [3].

## Case presentation

A 62-year-old independent woman with a medical history of systemic hypertension, non-insulin-dependent type 2 diabetes mellitus, obesity, obstructive sleep apnea syndrome, heart failure with preserved ejection fraction, asthma, and depression presented to the emergency department. She reported fatigue, reduced exercise tolerance, diffuse body aches, and unintentional weight loss of 10 kg over three months. Initial blood work, performed externally, revealed a hemoglobin level of 7.6 g/dL, prompting her referral.

On examination, she was pale, had a depressed effect, and a palpable mass in the left flank. Repeat labs showed microcytic hypochromic anemia (Hb 7 g/dL), leukocytosis, thrombocytosis, elevated C-reactive protein, and urinalysis suggestive of infection. A contrast-enhanced abdominopelvic CT scan revealed a simple cystic lesion in the left kidney (3.4 cm) with calcifications, enlarged lymph nodes, and a markedly enlarged uterus with multiple coalescent nodular lesions measuring up to 20×13 cm in the axial plane.

The patient was admitted for evaluation of constitutional symptoms, renal lesion, and uterine mass. Iron deficiency anemia was confirmed. A pelvic MRI showed a 4×3 cm T2-hypointense nodule in the left kidney with heterogeneous enhancement and a globally enlarged, globular uterus with multiple myometrial nodules. The most prominent nodule measured 13 cm. A thickened endometrium measuring 61 mm extended into the endocervical canal, which measured 36 mm.

Despite a broad diagnostic workup, the patient remained febrile with rising inflammatory markers during admission. Serologies (human immunodeficiency virus (HIV), hepatitis B virus (HBV), hepatitis C virus (HCV)) and blood cultures were negative. On day 3, she developed foul-smelling, pinkish vaginal discharge. Gynecology evaluation led to the initiation of metronidazole for suspected bacterial vaginosis.

A CT-guided biopsy of the renal mass confirmed an oncocytoma. The patient improved clinically and was discharged with outpatient follow-up.

**Figure 1:**
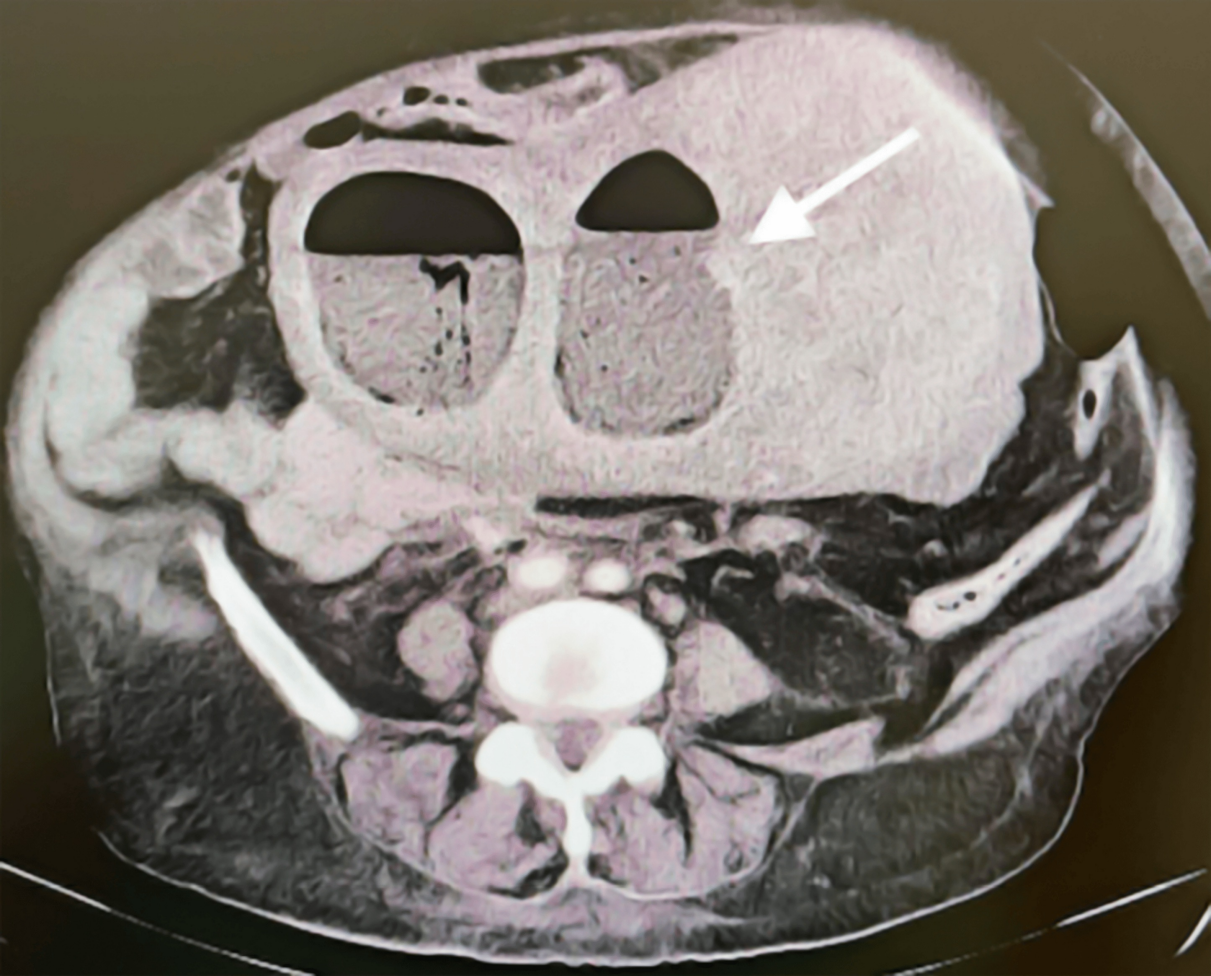
Abdomino-pelvic CT showing large intrauterine collection with air-fluid level and substantial solid-appearing nodular formation, consistent with a large hematopyometra.

Over the next four months, she had intermittent fever, persistent vaginal discharge, and required multiple empirical antibiotic courses (metronidazole, clindamycin, prulifloxacin, doxycycline) with no resolution. She returned to the ED with daily fever for a week, appearing pale, hypotensive, and reporting worsening symptoms. Labs showed Hb 8 g/dL and elevated CRP. Repeat abdominopelvic CT revealed a large intrauterine fluid collection with air-fluid level and enhancing rim, measuring 162 mm in length and 105×78 mm in axial plane, consistent with hematopyometra. A large adjacent solid mass (117×94×135 mm) suggested a possible myomatous origin (Figure 1).

The patient was transferred to gynecology. Drainage of the pyometra yielded purulent material positive for Escherichia coli and Enterococcus faecalis. She completed 21 days of meropenem. Endometrial biopsy revealed high-grade endometrioid adenocarcinoma with extensive necrosis and abscess formation. She underwent exploratory laparotomy with total hysterectomy, bilateral adnexectomy, and pelvic lymphadenectomy. Intraoperative pathology confirmed grade 3 endometrioid adenocarcinoma invading more than half of the myometrium.

## Discussion

This case illustrates an unusual presentation of endometrial adenocarcinoma manifesting as hematopyometra and persistent fever. The patient’s postmenopausal status and lack of recent sexual activity led to an underestimation of her gynecologic symptoms. Pyometra results from obstruction of cervical drainage, often secondary to tumors or fibrosis, and may be complicated by infection.

Although more commonly described in veterinary literature, pyometra is recognized in elderly women, particularly those with uterine pathology [2,4]. Clinical presentation is variable and may include vaginal discharge, fever, and lower abdominal pain. However, more than 50% of patients are asymptomatic [5]. Microbiological isolates often include polymicrobial flora such as *Escherichia coli* and *Bacteroides *spp. [2].

Risk factors include uterine neoplasms, cervical stenosis, estrogen excess, obesity, and prior radiation therapy [5]. In our patient, both postmenopausal status and obesity may have contributed to the pathogenesis. The underlying carcinoma likely caused endometrial thickening, impaired drainage, and superinfection. Mortality rates in untreated pyometra can reach up to 40% [5].

## Conclusions

Hematopyometra should be considered in postmenopausal women presenting with unexplained fever and uterine enlargement. Early imaging and gynecologic evaluation are crucial. This case underscores the importance of including gynecologic malignancies in the differential diagnosis of systemic inflammatory symptoms in elderly women.
